# *Piper betel* Compounds Piperidine, Eugenyl Acetate, and Chlorogenic Acid Are Broad-Spectrum Anti-*Vibrio* Compounds that Are Also Effective on MDR Strains of the Pathogen

**DOI:** 10.3390/pathogens8020064

**Published:** 2019-05-13

**Authors:** Erika Acosta-Smith, Nidia Leon-Sicairos, Sandeep Tiwari, Hector Flores-Villaseñor, Adrian Canizalez-Roman, Ranjith Kumavath, Preetam Ghosh, Vasco Azevedo, Debmalya Barh

**Affiliations:** 1 Programa Regional del Noroeste para el Doctorado en Biotecnología-FCQB, Universidad Autónoma de Sinaloa, Avenida de las Américas y Josefa Ortiz (Ciudad Universitaria), Culiacán Sin. 80030, Mexico; eaosmith@yahoo.com.mx; 2 CIASaP, Facultad de Medicina, Universidad Autónoma de Sinaloa. Cedros y Sauces, Fracc. Fresnos, Culiacán Sin. 80246, Mexico; nidialeon@uas.edu.mx (N.L.-S.); floreshectormanuel@gmail.com (H.F.-V.); canizalez@uas.edu.mx (A.C.-R.); 3 Departamento de Investigación, Hospital Pediátrico de Sinaloa, Blvd. Constitución y Donato Guerra S/N, Almada, Culiacán Sin. 80200, Mexico; 4 Laboratório de Genética Celular e Molecular, Programa de Pós-graduação em Bioinformática, Instituto de Ciências Biológicas (ICB), Universidade Federal de Minas Gerais, Av. Antonio Carlos 6627, Pampulha, Belo Horizonte, CEP 31270-901, Brazil; sandip_sbtbi@yahoo.com (S.T.); vascoariston@gmail.com (V.A.); 5 Department of Genomic Sciences, School of Biological Sciences, Central University of Kerala, Kasaragod 671121, India; rnkumavath@gmail.com; 6 Department of Computer Science, Virginia Commonwealth University, Richmond, VA 23284, USA; preetam.ghosh@gmail.com; 7 Centre for Genomics and Applied Gene Technology, Institute of Integrative Omics and Applied Biotechnology (IIOAB), Nonakuri, Purba Medinipur, West Bengal-721172, India

**Keywords:** antibiotics, multidrug resistances, *Vibrio cholera*, *Piper betel* compounds

## Abstract

The natural population of the aquatic environment supports a diverse aquatic biota and a robust seafood industry. However, this environment also provides an appropriate niche for the growth of pathogenic bacteria that cause problems for human health. For example, species of the genus *Vibrio* inhabit marine and estuarine environments. This genus includes species that are pathogenic to aquaculture, invertebrates, and humans. In humans, they can cause prominent diseases like gastroenteritis, wound infections, and septicemia. The increased number of multidrug resistant (MDR) *Vibrio* strains has drawn the attention of the scientific community to develop new broad-spectrum antibiotics. Hence, in this paper we report the bactericidal effects of compounds derived from *Piper betel* plants: piperidine, chlorogenic acid, and eugenyl acetate, against various strains of *Vibrio* species. The different MIC90 values were approximately in a range of 2–6 mg/mL, 5–16 mg/mL, 5–20 mg/mL, and 30–80 mg/mL, for piperidine, chlorogenic acid, and eugenyl acetate, respectively. Piperidine showed the best anti-*Vibrio* effect against the five *Vibrio* species tested. Interestingly, combinations of sub-inhibitory concentrations of piperidine, chlorogenic acid, and eugenyl acetate showed inhibitory effects in the *Vibrio* strains. Furthermore, these compounds showed synergism or partial synergism effects against MDR strains of the *Vibrio* species when they were incubated with antibiotics (ampicillin and chloramphenicol).

## 1. Introduction

*Vibrio* is a heterogeneous and polyphyletic genus with Gram-negative, curved-rod shaped, motile bacteria with high affinity for salinity and temperatures, fluctuating from 10 °C to 30 °C [1,2]. Several species of the genus are associated with infections like gastroenteritis, wound infection, and septicemia [3]. *Vibrio cholerae* O1 (classical O1 serotype strain), is the most important species responsible for cholera epidemics, and the species non-O1 serogroup *V. cholerae* O139 is the causative agent for gastroenteritis and extra-intestinal infections [4,5]. *V. cholerae* non-O1 also causes septicemia that leads to death [6,7]. *V. cholerae* serogroups Inaba and Ogawa belong to the classical and El Tor biotypes, and both serogroups were reported to be involved in cholera outbreaks [8,9,10]. *Vibrio parahaemolyticus*, a seawater bacterium, infects human through wounds or raw sea fish or seafood consumption, and causes inflammation of small intestine, diarrhea, cramping, and septicemia [11,12,13]. *Vibrio alginolyticus* and *Vibrio furnissii* are also seawater bacteria that cause superficial wound and ear infections (otitis media and otitis externa) [14] and diarrhea, respectively [15]. *Vibrio fluvialis* is uniquely associated with diarrhea outbreaks [16], and in rare cases, causes extra-intestinal infections such as hemorrhagic cellulitis with bacteremia, cerebritis, and peritonitis [17]. Although the infections caused by *Vibrio* species can be treated with various antibiotics, the multidrug resistant (MDR) strains emphasize the need to search for new broad-spectrum antibiotics to tackle the pathogens.

Historically, the shrubberies of *Piper betel* plant (family: *Piperaceae*) are used in Ayurvedic and folk medicine [18]. The crude extract is reported to be gastro-protective [19], with antimicrobial [20], anti-fungal [21], and anti-inflammatory [22] properties. However, the exact mechanism of the active compounds extracted from the betel leaf is still unclear.

Our group reported a set of seven compounds derived from the leaves of *Piper betel* plant (piperdardine, pinoresinol, guineensine, dehydropipernonaline, piperrolein B, eugenyl acetate, and chlorogenic acid), where some of these were previously proposed to be highly effective against a broad spectrum of *Vibrio* species. In a preliminary experimental work, 60 mM of piperdardine was shown to exhibit an equal growth inhibition effect to 100 µg/mL of chloramphenicol in *V. cholerae* O1 Inaba [23].

Here, we further report four *Piper betel* compounds (piperidine, eugenyl acetate, chlorogenic acid, and pinoresinol) that are effective against *V. cholerae* non-O1, *V. cholerae* O1 Ogawa, *V. cholerae* O1 Inaba, *V. parahaemolyticus*, *V. alginolyticus*, *V. furnissii*, and *V. fluvialis*. We also show that these *Piper betel* compounds are equally effective against MDR strains of the *Vibrio* species by acting in combination or in synergy with antibiotics [24].

## 2. Material and Methods

### 2.1. Bacterial Culture and Antibacterial Agents

*Vibrio cholerae* non-O1 Ogawa and *Vibrio cholerae* O1 Inaba were obtained from the National Institute of Diagnosis and Epidemiologic Reference (INDRE), Mexico. *Vibrio parahaemolyticus* TX2103 (CAIM 729) was obtained from the Collection of Aquatic Important Microorganisms (www.ciad.mx/caim), and had been isolated during the 1998 Texas (USA) outbreak [25,26]. *Vibrio cholerae* non-O1 (UIR22F), *Vibrio alginolyticus* (UIR22G1), *Vibrio furnissii* (UIR16A2), *Vibrio fluvialis* (UIR16A1), and *Vibrio parahaemolyticus* MDR (UIR10C4) strains were isolated by us [11,27,28,29,30]. The antimicrobial resistance (MDR) patterns of *Vibrio* spp. are given in Table 1A. Other authors have reported similar MDR patterns (Table 1B) [31,32,33,34,35,36,37]. Bacteria were cultured in Mueller–Hinton (MH) broth and maintained for 16–18 h in an incubating shaker at 37 °C to reach the logarithmic phase. The antibacterial compounds used in this study were purchased from Sigma Aldrich (St. Louis, MI, USA) and were dissolved in water (piperidine, 104094) or with 0.05% Tween 80 and 10% DMSO (chlorogenic acid, C3878; or eugenyl acetate, W246905; or pinoresinol, 40574). This solution (0.05% Tween 80 and 10% DMSO plus each compound) did not affect the bacterial cultures by itself during the experiments (not shown).

### 2.2. Evaluation of the Antibacterial Activity of Compounds on *Vibrio* spp.

Exponential phase bacteria were adjusted to an absorbance of 0.1 at 600 nm (approximately 10^7^ CFU/mL). The bactericidal activity of compounds was tested following two well-established methods. 

(1) Disk diffusion method; here 100 μL of the suspension of each *Vibrio* strain (containing 10^7^ CFU/mL) prepared from an overnight culture were used to seed each prepared and dried Mueller–Hinton agar plate. Then, commercial Sensi-Disks^TM^ (10 µg/mL ampicillin and 30 µg/mL chloramphenicol, purchased from BD) or sterile paper disc of 6 mm (filter paper mini Trans-Blot Bio-Rad Cat. No. 1703932) impregnated with the compound (piperidine in H_2_O, at concentrations of 1, 3, 7, and 10 mg/disk), were placed in MH agar plates, and then incubated at 37 °C for 24 h. Negative control was also prepared by impregnating paper disc with solvent (H_2_O) used to dissolve the piperidine. Finally, the antimicrobial activity was evaluated by measuring the inhibition diameter zone around the tested *Vibrio* strain [38]. The mean of the inhibition diameter zones for each antibacterial compound was determined as the average of three independent experiments. 

(2) For colony-forming units (CFU/mL) assay [39], approximately 10^5^ CFU/mL of bacterial suspensions were re-suspended in tubes containing MH broth either alone (control of bacterial growth) or with standard drug (30 µg/mL of chlorampenicol, control of bacterial inhibition), or with piperidine (1, 2, 3, 10 mg/mL dissolved in H_2_O), or with chlorogenic acid (5, 10, 15, 20, 25, or 30 mg/mL dissolved in 0.05% Tween 80 and 10% DMSO) or with eugenyl acetate (dissolved in 0.05% Tween 80 and 10% DMSO), or with pinoresinol (20, 30, 40, or 50 mg/mL dissolved in 0.05% Tween 80 and 10% DMSO), or with the solvents used (H_2_O or 0.05% Tween 80 and 10% DMSO). Tubes were maintained for 0, 20, 40, 60, and 80 min in an incubation shaker at 37 °C. The number of CFUs of viable bacteria was counted each time after inoculating the serial 10-fold dilutions from BHI broth onto BHI agar plates. 

### 2.3. Determination of Compound Minimum Inhibitory Concentrations against *Vibrio* spp.

The minimum inhibitory concentration (MIC) of compounds was determined by agar dilution method as described by the National Committee for Clinical Laboratory Standards (NCCLS). Briefly, the solution of compounds in serial two-fold concentrations were added into agar as follows: piperidine (0.5–16 mg/mL), eugenyl acetate (0.5–30 mg/mL), chlorogenic acid (0.5–40 mg/mL), and pinoresinol (0.5–50 mg/mL). The MIC was defined and the bacterial inocula were prepared as previously described, except that the final inocula of approximately 10^4^ CFU/spot of bacterial inoculum were applied to the plates and then incubated at 37 °C for 24 h. Quality control analyses of the methods were regularly performed for each test. The MIC for each compound also was calculated by the Disk diffusion method as has been described before. The MIC was recorded as the lowest concentration of antimicrobial agent with no visible growth.

### 2.4. Determination of the Inhibition Parameters of *Piper betel* Compounds in Combination with Compounds or Standard Drugs on *Vibrio* spp.

Various combinations of compounds plus *Piper betel* compounds, or compounds plus standard drugs were tested by the disk diffusion method and colony-forming unit assay (CFU/mL), as mentioned above. The concentrations of piperidine tested ranged from 0.5 to 16 mg/mL and for chlorogenic acid and eugenyl acetate from 0.5 to 40 mg/mL, and for the standard drugs chloramphenicol and ampicillin from 10, 15, and 30 µg/mL or 5, 10, and 50 µg/mL, respectively. The combinations for each strain were tested in triplicates. 

### 2.5. Determination of the Synergistic Activity of Compounds plus Antibiotics

To examine the effects of combinations of the different compounds on bacterial survival, or the synergistic activity of the compounds in combination with antibiotics, we used the checkerboard broth dilution method to determine the fractional inhibitory concentration index (FIC) [40]. This index is calculated according to the following formula: FIC of drug A (FIC A) = (MIC of drug A in combination)/(MIC of A); FIC of drug B (FIC B) = (MIC of drug B in combination)/(MIC of B). 

### 2.6. Statistical Analysis

All experiments were repeated at least twice in triplicate for confirmation of the results. Data were expressed as mean ± SEM, where SEM is the standard error of the mean. Data were compared using two-tailed Student’s *t*-test, and *p* < 0.05 was considered statistically significant.

Statistical analysis on synergy: The calculated FIC index was interpreted as synergistic (≤0.5), partial synergy (>0.5 but <1), indifferent (>1 but <4.0), or antagonistic (≥4.0) [40,41].

## 3. Results and Discussion

### 3.1. Bactericidal Activity of Piperidine on *Vibrio* spp.

**Figure 1:**
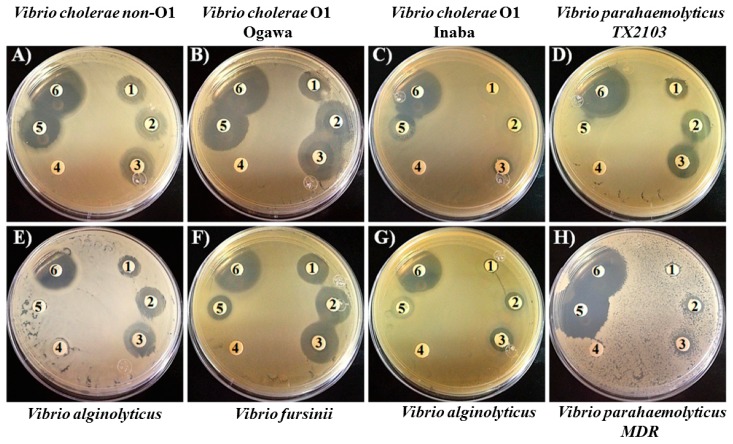
Antibacterial activity of piperidine against *Vibrio* spp. Mueller–Hinton agar plates were swabbed with Mueller–Hinton broth inoculated with *Vibrio* spp. and incubated to a turbidity of 0.5 McFarland standard; (**A**) *Vibrio cholerae* non-O1, (**B**) *Vibrio cholerae* O1 Ogawa, (**C**) *Vibrio cholerae* O1 Inaba, (**D**) *Vibrio parahaemolyticus* TX2103, (**E**) *Vibrio alginolyticus*, (**F**) *Vibrio furnissii*, (**G**) *Vibrio fluvialis*, and (**H**) *Vibrio parahaemolyticus* MDR. Impregnated filter paper with piperidine or commercially prepared antimicrobial agent disks were placed on the inoculated plates; (**1**) 3 mg/mL of piperidine, (**2**) 7 mg/mL of piperidine, (**3**) 10 mg/mL of piperidine, (**4**) H2O, (**5**) 50 µg/mL of ampicillin, and (**6**) 30 µg/mL of chloramphenicol. Note: The zones of inhibition around disks containing piperidine are concentration-dependent.

Piperidine exhibited bactericidal activity against all *Vibrio* species tested (Figure 1). By visualizing the inhibition zones, the best effects were on *V. cholerae* O1 Ogawa (B), *V. furnisii* (F), *V. cholerae* non-O1 (A), *V. parahaemolyticus* TX2103 (D), and *V. alginolyticus* (E). The inhibition zone was moderate in *V. parahaemolyticus MDR* (H), *V. fluvialis* (G), and *V. cholerae* O1 Inaba (C). The bactericidal effect of piperidine appeared during the first 24 hours of incubation and was concentration-dependent (Figure 1 disks 1–3 that correspond to 3, 7, and 10 mg/mL of piperidine, respectively). The anti-*Vibrio* effect of piperidine was better than the antibiotic ampicillin for ampicillin non-resistant *Vibrio* strains *V. cholerae* O1 Ogawa (B), *V. furnisii* (Figure 1F), and *V. fluvialis* (G). Considering the MDR spectrum of the tested strains (Table 1A,B), we observed that piperidine was effective on MDR strains of *V. cholerae* O1 Ogawa (B), *V. cholerae* O1 Inaba (C), *V. alginolyticus* (E), and *V. parahaemolyticus* MDR (H). 

Based on the disk diffusion method, we observed that 10 mg/mL of piperidine (disk 3) inhibited bacterial growth of *V. parahaemolyticus* TX2103, *V. alginolyticus*, *V. furnissiii*, and *V. fluvialis* strains, similar to a growth inhibitory effect of 30 µg/mL of chloramphenicol (Figure 1, A–H disk 6). Interestingly, these strains although susceptible to piperidine and chloramphenicol, were however resistant to 10–50 µg/mL of ampicillin, corroborating our previous determinations (Table 1). The compounds chlorogenic acid (10–30 mg/mL) and eugenyl acetate (10–40 mg/mL) also inhibited the growth of *Vibrio* spp. in similar conditions tested for the assays with piperidine (data not shown). However, intriguingly during the disk diffusion method the compounds chlorogenic and eugenyl acetate showed green and yellow pigmentation, respectively, on the inhibition zones (not shown). In this case, we decided to estimate the CFU/mL to corroborate the results explained above. By this method, we found that low concentration of the compounds were necessary to inhibit the growth of the *Vibrio* strains (Table 2), which was observed because this technique is more sensitive than the disk diffusion assay. The leaves of the *Piper betel* plant have long been in use in the local Indian system of medicine for its antioxidant and antimicrobial properties [42,43]. Some groups of researchers have reported the antimicrobial properties of *Piper betel* extracts [43,44]; however to the best of our knowledge, this is the first report of the antibacterial activity of *Piper betel* derivatives against *Vibrio* spp.

### 3.2. Determination of MICs against *Vibrio* spp.

All of the tested *Piper betel* compounds exhibited significant in vitro activity against approximately all *Vibrio* spp. In Table 2, the MICs of piperidine at which 90% of *Vibrio* spp. growth was inhibited (MICs90) were approximately in the range of 2–6 mg/mL, and those of chlorogenic acid were 5–16 mg/mL. The MICs90 of eugenyl acetate and pinoresinol were in the range of 5–20 mg/mL, and 30–80 mg/mL, respectively. Table 2 indicates results of only *V. cholerae* Inaba, *V. parahaemolyticus* TX 2103, *V. parahaemolyticus* O3:K6, *V. furnisii* (UIR16A2), and *V. fluvialis* (UIR16A1); however the compounds also affected other *Vibrio* spp. at the same concentrations (not shown). 

### 3.3. Antibacterial Activity of Mixtures of *Piper betel*-Derived Compounds against *Vibrio* spp.

According to our observations, the compound piperidine exhibited the best antibacterial activity in all *Vibrio* spp. When *V. cholera* Inaba, *V. parahaemolyticus* TX 2103, *V. parahaemolyticus* O6:K6, *V. furnisii*, and *V. fluvialis* were incubated with piperidine, chlorogenic acid, and eugenyl acetate at their MICs (Table 3), the bacterial growth was inhibited during the initial 24 and 36 h. Interestingly, when sub-inhibitory concentrations of piperidine (0.5–2.0 mg/mL), chlorogenic acid (1.0–2.0 mg/mL), and eugenyl acetate (0.5–2.0 mg/mL) were combined, these three compounds (0.5–3.0 mg/mL) were able to inhibit the growth of all *Vibrio* spp. (Table 2). The inhibitory effect persisted for more than 24 h with no noticeable regrowth. 

### 3.4. Antibacterial Activity of Piperidine in Combination with Ampicillin or Chloramphenicol against *Vibrio* spp.

**Figure 2:**
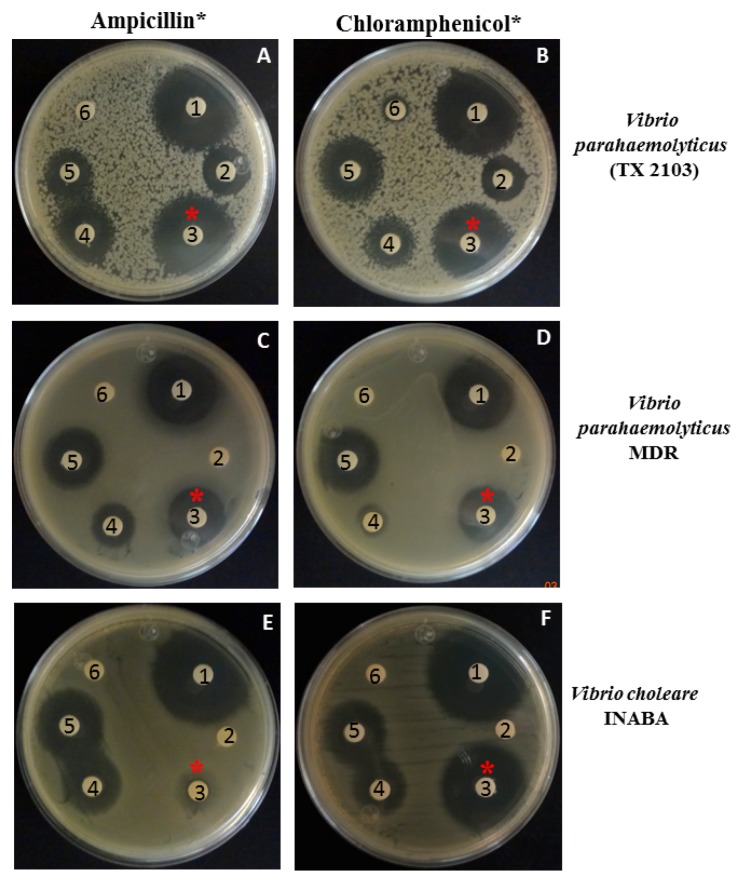
Antibacterial activity of piperidine in combination with ampicillin or chloramphenicol against *Vibrio* spp. Mueller–Hinton agar plates were swabbed with Mueller–Hinton broth inoculated with *Vibrio* spp. and incubated to a turbidity of 0.5 McFarland standard; (**A**) and (**B**) *Vibrio parahaemolyticus* TX2103; (**C**) and (**D***) Vibrio parahaemolyticus* multi-drug resistant (MDR); and (**E**) and (**F**) *Vibrio cholerae* Inaba. Commercially prepared antimicrobial agent disks were placed on the inoculated plates with (**1**) 30 µg/mL of chloramphenicol (control of bacterial growth inhibition), (**2**) 50 µg/mL of ampicillin, or (**3**) 1 mg/mL of piperidine plus 2.5 µg/mL of ampicillin ((**A**), (**C**), and (**E**), respectively), and/or impregnated filter paper with the combination of 1 mg/mL of piperidine plus 7 µg/mL chloramphenicol ((**B**), (**D**) and (**E**), respectively), (**4**) 1 mg/mL of piperidine, (**5**) 4 mg/mL of piperidine, or (**6**) H_2_O.

The antibacterial effect of compounds mixed in combinations against Vibrio was also tested. In these experiments, we used combinations of compounds in Vibrio strains that were resistant to antibiotics, *V. parahaemolyticus* MDR, *V. parahaemolyticus* TX2103 and *V. cholerae* Inaba. In the results, (Figure 2), at concentrations of 1 and 3 mg/mL of piperidine (Panel A, C, and E: disks 4, and 5, respectively), an inhibition zone was observed (more visible at 4 mg/mL piperidine), but when 1 mg/mL of piperidine was added in the filter in combination with 2.5 µg/mL of ampicillin, a clear inhibition zone was observed in *V. parahemolyticus* MDR and *V. parahaemolyticus* (panels A and C, disk 3), indicating that in combination the antibacterial activity is better. In disk 2 approximately 2.5 µg/mL of ampicillin were added; however this was not effective in inhibiting the growth (Panel A, C, and E: disks 2). 

Moreover, in these strains, the combination of 1 mg/mL of piperidine and 7 µg/mL of chloramphenicol (panels B, D, and F, respectively, filter 3) showed a inhibition zone, similar to those obtained in filters impregnated with 30 µg/mL of chloramphenicol (disk 1 in all panels) used as control of inhibition. Filters or disk 6 correspond to H_2_O (used to dissolve piperidine) and ampicillin, respectively. The present data were corroborated by CFU/mL counts where we observed similar effects. 

In the case of chlorogenic acid, a range of 6–20 mg/mL had antibacterial activity; however if 3 mg/mL of this compound were combined with 15 µg/mL of chloramphenicol or 10 µg/mL of ampicillin, we observed antibacterial activity. Eugenyl acetate inhibited the bacterial growth of *Vibrio* spp. at concentrations ranging from 6 to 20 mg/mL. When sub-inhibitory concentrations of this compound (3 and 10 mg/mL) were added in combination with 10 µg/mL of chloramphenicol or ampicillin, the inhibition zones were similar to those obtained with 30 or 50 µg/mL of chloramphenicol or ampicillin, respectively (Table 3). 

Additionally, we evaluated the possible synergistic effect of the compounds in the presence of antibiotics against three different *Vibrio* strains. In the test results, the FIC index of piperidine in combination with chloramphenicol or ampicillin ranged from 0.45 to 0.83 against the three different *Vibrio* spp. tested (Table 3). Piperidine induced an increase in the activity of both chloramphenicol and ampicillin and had partial synergistic effects with chloramphenicol and ampicillin in all the strains tested; however in combination with ampicillin it exhibited synergistic effects against *V. parahaemolyticus* MDR, and *V. cholera* O1 Inaba. 

On the other hand, chlorogenic acid induced an increase in the activity of both antibiotics in all the *Vibrio* strains; it showed partial synergism ranging from 0.35 to 1 FIC index but in combination with ampicillin in *V. parahaemolyticus* MDR it demonstrated synergistic effect (Table 3). Similar results were observed with eugenyl acetate and antibiotics in the three *Vibrio* strains. In addition, the combination with ampicillin presented synergistic effects against *V. cholera* O1 Inaba (Table 3).

Nowadays, drug-resistant bacterial infections cause substantial mortality and morbidity in patients, and this is due to the spread of bacterial strain antibiotic resistance. This has become a significant global public health concern [45]. Original approaches to combat multidrug resistant microorganisms are currently lacking, and adversely affect various areas of clinical medicine such as the care of critically and chronically ill, transplantation medicine, and surgery etc. Hence, there is an urgent need for effective drugs to prevent and combat opportunistic pathogens. The World Health Organization has identified MDR bacteria as one of the top three threats to human health [45]. One approach to combating MDR infections is the combination of two or more antimicrobial compounds of natural or synthetic origin with different modes of action. This is an attractive alternative, leading to the search for new compounds which have potential against MDR pathogens; however, we must investigate their modes of action, efficacy, and safety in animal models and finally in clinical trials. 

In the context of the mechanism of action of the compounds, our results lead to the speculation that the mechanism is based on an alteration in bacterial membrane permeabilization, as the different *Vibrio* species tested here showed different susceptibilities to the compounds. *Vibrio* spp. has different virulence factors, serotypes etc., because of which the modes of action and target sites can be different. It is important to denote that the antibiotics used in this work were chosen because our clinical isolates of *Vibrio* spp. were resistant to ampicillin and also because chloramphenicol is used to treat *Vibrio cholerarae* infections. Altogether our data indicate that these compounds have strong growth inhibitory effects on various *Vibrio* spp. These compounds have potential therapeutic effects, and also exerted a convincing antibacterial effect in different proportions by themselves or in combination with each other or with the antibiotics used.

## Figures and Tables

**(A):** Antimicrobial resistance pattern of *Vibrio* spp. used in this work.

*Vibrio* Strains	Resistance Pattern							
	Tetracycline	Chloramphenicol	Ampicillin	SXT	Cefotaxime	Gentamicin	Ciprofloxacin	Nalidixic Acid
*V. cholerae* O1 Inaba	S	S	S	S	S	**R**	**R**	**R**
*V. cholerae* O1 Ogawa	**R**	S	S	S	**R**	S	S	R
*Vibrio fuvialis*	I	S	**R**	I	**R**	S	S	I
*Vibrio furnissii*	S	S	S	S	S	S	S	S
*V. parahaemolyticus* MDR	**R**	S	S	**R**	**R**	S	**R**	S
*V. parahaemolyticus* TX2103	S	S	**R**	S	S	S	S	S
*V. vulnificus*	**R**	S	S	S	I	I	**R**	S
*V. alginolyticus*	**R**	S	**R**	S	**R**	S	S	R
*V. cholerae* non-O1 serotype and toxigenic	S	S	S	S	S	S	**R**	S

S (Sensible), R (Resistant) I (intermeddle).

**(B):** Multidrug resistant (MDR) spectrum of *Vibrio* spp.

*Vibrio* Species	MDR Drugs	References
*Vibrio cholerae* O1 (Inaba and Ogawa serotype)	Ampicillin, polymyxin B, nalidixic acid, co-trimoxazole, norfloxacin, ciprofloxacin, doxycycline, gentamicin, chloramphenicol	Balaji et al. 2013
*V. cholerae* serogroup O1 Ogawa and El Tor	Co-trimoxazole, nalidixic acid, tetracycline, azithromycin, fluoroquinolones	Tran et al. 2012
*V. cholerae* non-O1, non-O139 serogroups	Norfloxacin and ciprofloxacin	Krishna et al. 2006
*V. parahaemolyticus*	Ampicillin and streptomycin, followed by carbenicillin, cefpodoxime, cephalothin, colistin, amoxycillin, nalidixic acid, tetracycline, chloramphenicol, and ciprofloxacin	Sudha et al. 2012
*V. alginolyticus*	Ampicillin, tetracycline, trimethoprim, and rifampin	Oh et al. 2011
*Vibrio fluvialis*	14 antibiotics including neomycin, co-trimoxazole, nalidixic acid, trimethoprim, ampicillin, kanamycin, ciprofloxacin, streptomycin, sulfisoxazole, chloramphenicol, norfloxacin	Rajpara et al. 2009; Mohanty et al. 2012

**Table 2:** Individual and synergistic antimicrobial activity of compounds.

Compounds	MIC (mg/mL) ^a^									
	MICS of Each Compound Incubated in the Cultures					MICS of Each Compound When All Were Incubated in the Cultures				
	*Vibrio cholerae* INABA	*Vibrio parahaemolyticus* TX 2103	*Vibrio parahaemolyticus* O3:K6	*Vibrio furnisii*	*Vibrio fluvialis*	*Vibrio cholerae* INABA	*Vibrio parahaemolyticus* TX 2103	*Vibrio parahaemolyticus* O3:K6	*Vibrio furnisii*	*Vibrio fluvialis*
Piperidine mg/mL	2 ± 0.5	2 ± 0.5	6.5 ± 0.5	4 ± 0.5	2 ± 0.8	0.6 ± 0.4	0.6 ± 0.3	1.7 ± 0.5	1 ± 0.6	1 ± 0.4
Chlorogenic acid mg/mL	5.5 ± 0.5	5.5 ± 1	16 ± 4	2 ± 0.5	6.5 ± 0.5	1.8 ± 0.2	1.8 ± 0.2	2 ± 0.8	1 ± 0.4	2 ± 0.4
Eugenyl acetate mg/mL	20 ± 4	5.5 ± 0.5	≥16 ± 6	6.5 ± 0.5	6.5 ± 1	2 ± 0.25	0.5 ± 0.25	2 ± 0.8	2 ± 0.6	2 ± 0.8
Pinoresinol mg/mL	≥30	≥30	≥30	-	-	-	-	-	-	-

^a^ Minimum inhibitory concentrations (MICs) at which 90% of bacterial cultures are inhibited; respectively.

**Table 3:** Determination of the synergist effect of compounds and antibiotics.

Strains	Agent	MIC		FIC Index *	Outcome *
		Alone	Combination		
***Vibrio parahaemolyticus*** **MDR**	Chloramphenicol (µg/mL)	30	22.5	0.75	Partial synergy
	Piperidine (mg/mL)	4	3		
	Ampicillin (µg/mL)	50	10	0.45	**Synergy**
	Piperidine (mg/mL)	4	1		
	Chloramphenicol (µg/mL)	30	15	1	Partial synergy
	Chlorogenic acid (mg/mL)	20	10		
	Ampicillin (µg/mL)	50	10	0.35	**Synergy**
	Chlorogenic acid (mg/mL)	20	3		
	Chloramphenicol (µg/mL)	30	10	0.83	Partial synergy
	Eugenyl acetate (mg/mL)	20	10		
	Ampicillin (µg/mL)	50	10	0.7	Partial synergy
	Eugenyl acetate (mg/mL)	20	10		
***Vibrio parahaemolyticus*** **TX2103**	Chloramphenicol (µg/mL)	30	11.25	0.75	Partial synergy
	Piperidine (mg/mL)	4	1.5		
	Ampicillin (µg/mL)	≥100	10	0.83	Partial synergy
	Piperidine (mg/mL)	4	2		
	Chloramphenicol (µg)	30	15	1	Partial synergy
	Chlorogenic acid (mg/mL)	6	3		
	Ampicillin (µg/mL)	≥100	10	0.6	Partial synergy
	Chlorogenic acid (mg/mL)	6	3		
	Chloramphenicol (µg/mL)	30	15	1	Partial synergy
	Eugenyl acetate (mg/mL)	6	3		
	Ampicillin (µg/mL)	≥100	10	0.6	Partial synergy
	Eugenyl acetate (mg/mL)	6	3		
***Vibrio*** ***cholerae* O1 INABA**	Chloramphenicol (µg/mL)	30	7.5	0.5	**Synergy**
	Piperidine (mg/mL)	4	1		
	Ampicillin (µg/mL)	50	10	0.45	**Synergy**
	Piperidine (mg/mL)	4	1		
	Chloramphenicol (µg/mL)	30	10	0.83	Partial synergy
	Chlorogenic acid (mg/mL)	6	3		
	Ampicillin (µg/mL)	50	10	0.7	Partial synergy
	Chlorogenic acid (mg/mL)	6	3		
	Chloramphenicol (µg/mL)	30	10	0.58	Partial synergy
	Eugenyl acetate (mg/mL)	20	5		
	Ampicillin (µg/mL)	50	10	0.45	**Synergy**
	Eugenyl acetate (mg/mL)	20	5		

* The fractional inhibitory concentration (FIC) index was interpreted as synergy at ≤0.5, partial synergy at >0.5 but <1.0, indifferent at >1.0 and <4.0, and antagonistic when values were ≥4.0.
